# fMRI Study of Social Anxiety during Social Ostracism with and without Emotional Support

**DOI:** 10.1371/journal.pone.0127426

**Published:** 2015-05-22

**Authors:** Yoshiko Nishiyama, Yasumasa Okamoto, Yoshihiko Kunisato, Go Okada, Shinpei Yoshimura, Yoshihiro Kanai, Takanao Yamamura, Atsuo Yoshino, Ran Jinnin, Koki Takagaki, Keiichi Onoda, Shigeto Yamawaki

**Affiliations:** 1 Department of Psychiatry and Neurosciences, Graduate School of Biomedical and Health Sciences, Hiroshima University, Hiroshima, Japan; 2 Department of Psychology, School of Human Sciences, Senshu University, Kawasaki, Japan; 3 Department of Psychology, Faculty of Psychology, Otemon Gakuin University, Osaka, Japan; 4 Department of Human Science, Faculty of Liberal Arts, Tohoku Gakuin University, Sendai, Japan; 5 Department of Neurology, Shimane University, Izumo, Japan; Central Institute of Mental Health, GERMANY

## Abstract

Social anxiety is characterized by an excessive fear of being embarrassed in social interactions or social performance situations. Emotional support can help to decrease or diminish social distress. Such support may play an important role at different points of social interaction. However, it is unclear how the beneficial effects of social support are represented in the brains of socially anxious individuals. To explore this, we used the same paradigm previously used to examine the effects of emotional support on social pain caused by exclusion. Undergraduates (n = 46) showing a wide range of social anxiety scores underwent functional magnetic resonance imaging (fMRI) while participating in a Cyberball game. Participants were initially included and later excluded from the game. In the latter half of the session in which participants were excluded, they were provided with supportive messages. In line with our previous work, we found that social exclusion led to increased anterior cingulate cortex (ACC) activity, whereas emotional support led to increased left dorsolateral prefrontal cortex (DLPFC) activity. Despite validation of the paradigm, social anxiety was not associated with increased ACC activity during social exclusion, or during perceived emotional support. Instead, fear of negative evaluation as assessed by the Brief Fear of Negative Evaluation (BFNE) scale showed positive associations with left DLPFC activation while receiving emotional support, compared to while being socially excluded. The more socially anxious an individual was, the greater was the left DLPFC activity increased during receipt of messages. This suggests that highly socially anxious people still have the ability to perceive social support, but that they are nevertheless susceptible to negative evaluation by others.

## Introduction

Social anxiety is characterized by an excessive fear of being embarrassed during social interactions. This embarrassment stems from being scrutinized, negatively criticized, or excluded [1, 2]. The severity of social anxiety is continuously distributed [3–7] and an excessive form of the condition has been labeled social anxiety disorder (SAD) [8]. Certain clinical features of individuals with high social anxiety have been described, including poor social relationships [9, 10], difficulties with emotional regulation [11], and sensitivity to the perceived threat of social isolation [10, 12, 13]. People with high social anxiety also have poor quality and fewer intimate social interactions with others, including basic acquaintances and intimate partners [10, 14]. Certain studies investigating social ostracism have employed a ball catching game paradigm named Cyberball, in which participants are virtually ostracized [15–18]. In Cyberball, participants play catch with two other players whose actions are in fact computer generated. The two computer-generated players toss the ball to the participant at the beginning of the game and after a while they may continue to do so, or may not throw the ball to the participant at all, or do so only infrequently. Social ostracism evokes physiological [19] as well as psychological reactions [15–17], and reactivity to exclusion simulated by the Cyberball computer task prospectively predicted social anxiety 2 months later [20].

The Cyberball task has been administered to socially anxious individuals, and when they are excluded, they show reactions distinct from those of socially non-anxious persons [21, 22]. Excluded socially anxious people show lower need scores, including belonging (e.g., “I felt like an outsider”), self-esteem (e.g., “I felt good about myself”), control (e.g., “I felt like I had control over the course of the interaction”), and meaningful existence (e.g., “I felt nonexistent”), than socially non-anxious persons [21], indicating that socially excluded individuals, especially those with high social anxiety, experience greater need-threat. Oaten et al. (2008) [22] also used the Cyberball paradigm and showed disordered self-regulation in people with high social anxiety.

In order to examine the neural responses underlying the effect of ostracism, neuroimaging studies have been conducted during Cyberball. It has been reported that healthy people show increased anterior cingulate cortex (ACC) and insula activity when they are excluded from getting the ball [16, 17]. Emotional support can frequently help to diminish social distress. Such support may play an important role at different points in the chain of events that begins with a potential stressor and culminates in physiological stress [23]. Regarding emotional support, our previous study demonstrated that when healthy participants got emotionally-supportive messages while they were being excluded from getting the ball, they showed both decreased social distress and decreased ACC activation compared to when they were socially excluded without supportive messages [17]. In addition, left dorsolateral prefrontal cortex (DLPFC) activation was negatively correlated with participants’ subjective feelings of social distress: Increased DLPFC activation corresponded to increased beneficial effects of social support on subjective social pain [17]. However, to the best of our knowledge, previous studies have not investigated brain areas associated with social anxiety, either while people are excluded, or while they are being emotionally supported.

The present experiment was conducted using the Cyberball paradigm to examine particular neural responses associated with social anxiety while people with a broad range of social anxiety scores were ostracized and were provided with emotional support while being ostracized. We investigated (1) if social anxiety is positively correlated with ACC activation while people are excluded from a virtual ball-tossing game, and (2) if emotional support was less effective in ameliorating the subjective distress of more socially anxious people while they were being ostracized, by examining if DLPFC region activation is negatively correlated with increased subjective social distress and social anxiety.

## Method

### Ethics Statement

The Ethics Committee of Hiroshima University approved the present study and all participants signed a written informed consent form.

### Participants and questionnaires

Forty nine right-handed undergraduates took part in the study. Three of them were excluded from analyses because of protocol malfunction, and 46 people (29 women; mean age = 19.85 years) were ultimately included in the final analysis. Of these, 37 participants had no current Axis I disorders, whereas 9 participants met criteria for SAD based on the Structured Clinical interview for DSM-IV, Axis I (SCID). Participants also completed the Brief Fear of Negative Evaluation Scale (BFNE) [24]. The BFNE is a brief version of the original Fear of Negative Evaluation Scale (FNE) [25] that assesses the interaction-related anxiety subtype, which is part of the social anxiety spectrum [26]. People with high FNE show a more negative perception of their own actions as the anxiety level in a situation increases [27]. BFNE scores are highly correlated with original FNE scores [24] and were found to provide more information than the original FNE [28]. The BFNE also has the practical advantage of brevity, and has become a frequently used instrument in social anxiety research with non-clinical populations [29–31]. The mean BFNE score for patients with social phobia is reported to be 51.5 points (standard deviation (SD) = 7.3), with a range between 30.0 to 60.0 points, whereas the mean score of a community sample is 29.2 points (SD 8.2), with a range between 16.0 to 52.0 points [32]. As the BFNE scores in the present study ranged from 18 to 57 points (median = 40), with the mean score being 39.65 points (SD = 11.03), participants in the present study showed a wide range of social anxiety. Participants also completed a 12-item measure of social distress [15] which assesses participants’ subjective experience of social distress during the Cyberball task, including self-esteem (“I felt liked”), belongingness (“I felt rejected”), meaningfulness (“I felt invisible”), and control (“I felt powerful”) [15–17, 22]. As in previous studies [15–17, 22], we used these 4 items for present analyses. Each item is rated from 1 to 9, with total scores therefore ranging from 4 to 36. It has been reported that this measure of subjective experience has acceptable reliability and validity [22].

### fMRI session

**Fig 1:**
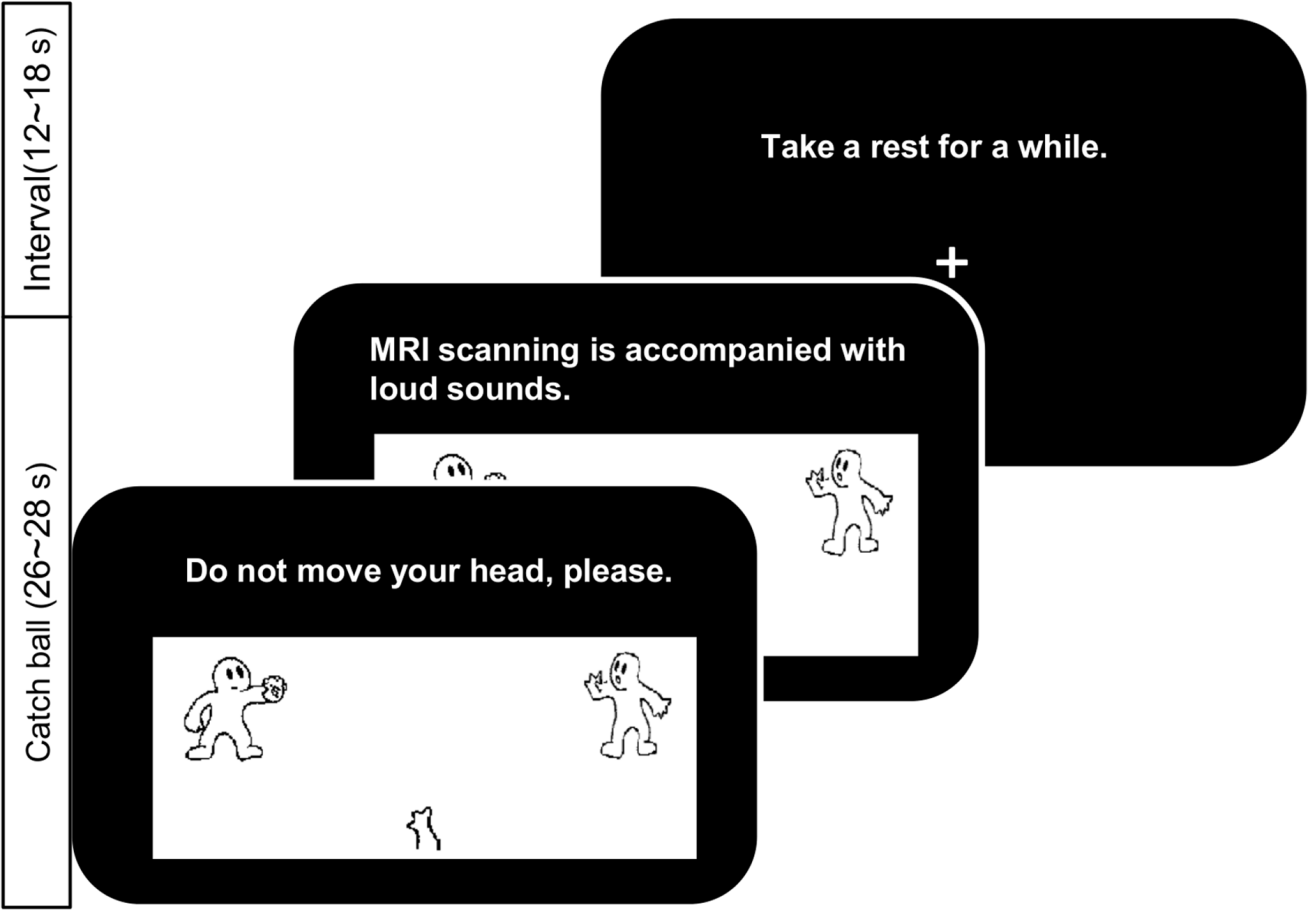
Cyberball Paradigm. Each block was composed of a ball catching phase for 26~28 seconds, with messages provided on the top center of the screen. Two messages and intervals of 12~18 seconds were provided for each block. There were five blocks in each condition.

An experimental manipulation of social exclusion (ostracism) was conducted using the Cyberball task, as modified by Onoda et al., (2009, 2010); see Fig 1 [15, 33]. Participants were initially told that the experimenters were interested in the neural mechanisms that underlie mental visualization ability, and that they would be playing a game of catch with two other players (actually computer generated), while being connected via the Internet. The two other players, whose photographs were shown to the participants before the fMRI session, were of the same gender and similar age as the participants. The photographs of the two supposed players were obtained from the SOFTPIA JAPAN database (the service has since been terminated), by selecting 20 neutral faces of people in their twenties (10 of each gender). Twenty-three graduate students then rated the faces on three aspects: Preferences, congeniality, and attractiveness. For each gender, four photographs with median ratings on these aspects were selected and used for the experimental sessions, to be used in pairs. Two combinations of two photographs were selected for each gender and were counter balanced.

Participants saw a ball, cartoon images representing the two virtual computer players on the left and right sides of the screen, an arm representing the participant on the lower central portion of the screen, and a message at the top of the screen (Fig 1). The virtual players automatically threw the ball to each other or to the participant. The participant was free to decide who would next receive the ball, and to throw the ball by pressing a button on a button box. The ball was thrown 9~12 times per block. Participants were also told that when one of the other players caught the ball, they were to push the button on the same side of the button box as the player who caught the ball. Throughout the ball-tossing game, messages were displayed. Participants were told to pay attention to messages that were supposedly displayed on the screen by experimenters who were watching the ball-tossing game in a separate room. In fact, the messages had been preprogrammed. A message was presented for the first half of a block, and then another message was shown for the second half of the block. Therefore, two messages per block (10 messages per condition) were shown to the participants.

The fMRI task in the Cyberball paradigm consisted of four conditions, and each condition contained five blocks with a duration of 26~28 seconds per block and a 12~18-second rest periods between blocks. Because the paradigm was self-advancing for each participant, block length varied slightly across individuals. The first condition was the control condition (CON). In CON, participants were told that experimenters had to confirm the connection to the Internet through the participants’ button push. Therefore, participants knew that they would not be thrown a ball during the control condition. The next condition was the social inclusion condition (IN), during which participants received three or four throws per block. The ratio of ball tosses received by participants varied between 30%-35% across the five inclusion blocks. The third condition was the social exclusion condition (EX), during which participants received no ball throws. Participants were told that after they received a message to start playing catch with three people at the end of the CON condition, the catch game would start and continue until the end of the Cyberball task, such that they could expect that balls would be thrown to them throughout the Cyberball task. On the other hand, participants were also told that when one of the other players caught the ball, they had to push the button on the same side of the button box as the player who caught the ball. Although participants were anticipating ball throws throughout the Cyberball task, they were never thrown the ball during the EX condition. Therefore, participants had to push the button on the same side of the button box as the player who caught the ball, while in fact anticipating that they would be thrown balls during the EX condition. As a result, the difference between CON and EX was that participants were aware in advance that they were not supposed to be thrown the ball (CON), or they were not aware of this (EX). In CON, IN, and EX, messages displayed on the screen consisted of experimental instructions: For example, “The button box is under validation,” “Intervals are included in a certain period of time,” “Do not sleep, please.” The last condition was the social support condition (SUP); this condition and EX were identical, except for the messages, which were caring statements instead of experimental instructions [17]. Emotionally-supportive messages were chosen from 17 messages on the basis of 23 graduate students’ ratings. The ratings were based on understanding the participants’ feelings, taking a hopeful view of the situation, and encouragement to remain in the socially excluded situation. The ten highest-rated messages were selected and displayed on the screen during SUP. Examples of emotionally-supportive messages included “We are sorry for making you feel terrible,” and “I know it was unpleasant for you to be excluded.”

Upon completion of the virtual game, participants retrospectively completed questionnaires outside of the MRI that assessed their subjective experiences [15] during each condition of the Cyberball task, with the exception of CON. As we had to make participants believe that they were playing catch with actual people instead of computer generated players, we did not assess social distress during CON [16]. Instead, we assessed participants’ subjective experiences before the fMRI session as a baseline. Since we examined the imaging data for contrasts between the conditions, as will be described subsequently in the fMRI data acquisition section, we subtracted the numerical value of participants’ subjective experiences at baseline from those during the three analyzed conditions (IN, EX, and SUP). At the end of the procedure, participants were fully debriefed.

### fMRI data acquisition

A Symphony 1.5 tesla scanner (Siemens AG, Symphony, Tokyo, Japan) was used to acquire imaging data. A time-course series of 297 volumes per participant was acquired with echo planar imaging (EPI) sequences (repetition time (TR) = 3000 ms, echo time (TE) = 40 ms, field of view (FOV) = 256 mm, matrix size = 64 x 64, 30 slices, 4 x 4 x 4 mm voxel dimensions, flip angle =. 90)). Functional scans lasted 14 min and 57 s, including a pre-baseline interval (15 s). After functional scanning, structural scans were acquired using T1-weighted gradient echo pulse sequences (TR = 12 ms, TE = 4.5 ms, FOV = 256 mm, flip angle =. 20), which facilitated localization.

### Data analysis

Image processing and statistical analyses were carried out using Statistical Parametric Mapping (SPM8) software (Wellcome Department of Cognitive Neurology, London, UK) under Ubuntu Linux 10.04. The first five volumes of the fMRI run were discarded because the MRI signals were unsteady. All EPI images were realigned to the first volume, slice timing correction was performed for each set of functional volumes, spatially normalized to a standard template based upon the Montreal Neurological Institute (MNI) reference brain, and smoothed using an 8-mm full width at half maximum Gaussian kernel. To perform image data analysis, a whole-brain voxel-by-voxel multiple linear regression model was employed at the individual participant level. Four regressors for each condition (CON, EX, IN, and SUP) were modeled with a canonical hemodynamic response function. The realignment parameters were also included in the model as a covariate of no interest. We created the following two corresponding contrasts for the first-level analysis for each participant to isolate brain circuits related to (1) social exclusion and (2) social support: (1) EX–IN and (2) SUP–EX. These individual contrast images were used at the whole-brain group-level, random-effects analyses. First, one sample *t*-tests were performed to assess the overall effect of each contrast and to see if the present sample with the current experimental paradigm showed acceptable neural activity consistent with previous healthy samples. Second, regression analysis using continuous social anxiety as measured by the BFNE was performed to assess the effect of social anxiety on each contrast. We decided on a cut-off threshold that would minimize the risk of Type II errors [34]. However, we also had to reduce Type I error risk. Therefore, we decided to employ a cut-off threshold of *p* < 0.005 (without a correction for multiple comparisons) with a cluster size of k > 30, after Choi et al. (2009) [35], instead of a cluster size of k > 10 as recommended by Lieberman et al. (2009) [34]. Additionally, we reported results using Region-of-Interest (ROI) analyses as supporting information, in order to confirm the overlap between reported regions and those of previous studies. A ROI for the ACC was defined as a 10mm-sphere centered on 10, 32, and -10, based on peak voxels identified in our previous study during an EX to IN comparison. Another ROI was defined for the DLPFC as a 20mm-sphere centered on -34, 24, and 22, based on peak voxels identified in our previous study during a SUP to EX comparison. In these ROI analyses a stricter significance level of p<.05 for family wise error (FWE) corrected for the small volume based on our previous study [17] was used. We created EX-IN contrast to isolate brain circuits related to social exclusion, and we created a SUP-EX contrast to isolate brain circuits related to social support using this threshold. If brain regions that are not associated with social exclusion or social support correlated with social anxiety as measured via the BFNE, it would be difficult to interpret such an association with consideration to social anxiety. Therefore, we focused on brain regions that are associated with social support or social exclusion and are also known to be modulated by social anxiety.

Activated clusters were localized using Anatomical Automatic Labeling for SPM8 ver. 1 (http://www.cyceron.fr/web/aalanatomicalautomaticlabeling.html). The ROIs for the ACC and DLPFC were defined using the WFU Pickatlas (http://www.fmri.wfubmc.edu/download.htm). Behavioral data was analyzed using one-way repeated measures ANOVA with the Statistical Package for the Social Science (SPSS) version 20.0.0. Averaged contrast values estimated during SUP compared to EX in the active cluster from the regression analysis was extracted using MarsBar (http://marsbar.soureeforge.net/).

## Results

### Behavioral results

**Fig 2:**
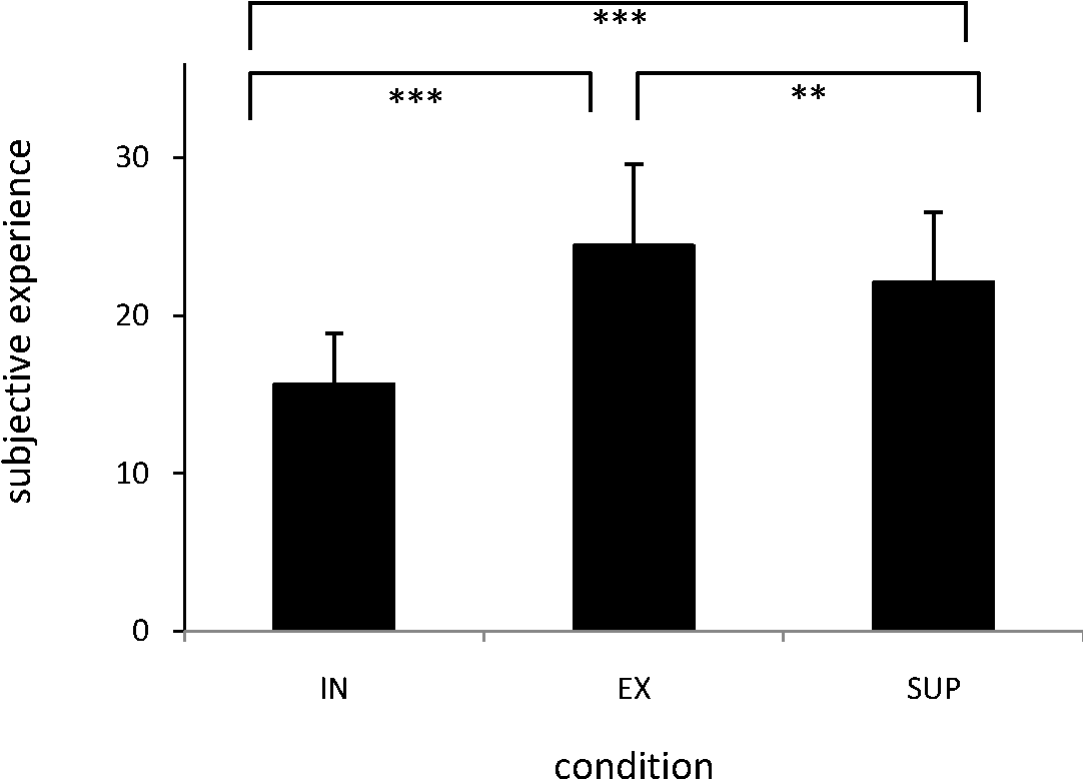
Subjective experiences of social distress during each condition. The values for subjective experience of social distress were: (1) during participation in the virtual ball-tossing game (IN, mean value = 15.70, standard deviation (SD) = 3.18, 95% confidence interval (CI) 14.75–16.64), (2) during exclusion from getting the ball (EX, mean value = 24.48, SD = 5.14, 95% CI 22.95–26.01), and (3) during supportive messages (SUP, mean value = 22.15, SD = 4.41, 95% CI 20.84–23.46). There were significant main effects of condition: Social pain was higher during EX and during SUP than during IN, and lower during SUP than during EX. IN = the social inclusion condition, EX = the social exclusion condition, SUP = the social support condition. *** *p* < 0.001, ** *p* < 0.005.

We performed a one-way repeated measures analysis of variance (ANOVA) to analyze participants’ subjective experience of social distress during participation in a ball-tossing game (IN), during exclusion from a ball-tossing game (EX), and when being offered emotional support (SUP) while being excluded from the game. Participants’ self-reported social distress levels are shown in Fig 2. There was a significant main effect of condition, *F* (2, 90) = 52.15, *p* < 0.001, η^2^ = 0.537. Bonferroni post-test analysis indicated that the subjective social distress during IN was lower than during EX (*p* < 0.001, Hedges’s g (g) = 0.47), as well as lower than that during SUP (*p* < 0.005, g = 0.34). Moreover, social distress during EX was higher than during SUP (*p* < 0.005. g = 0.12). There were no significant positive or negative correlations between subjective social distress and self-reported social anxiety for the EX-IN or SUP-EX comparisons (EX-IN: *r* = 0.232, *p* = 0.121, SUP-EX: *r* = -0.082, *p* = 0.587).

### Neural activity during social exclusion and social support

**Fig 3:**
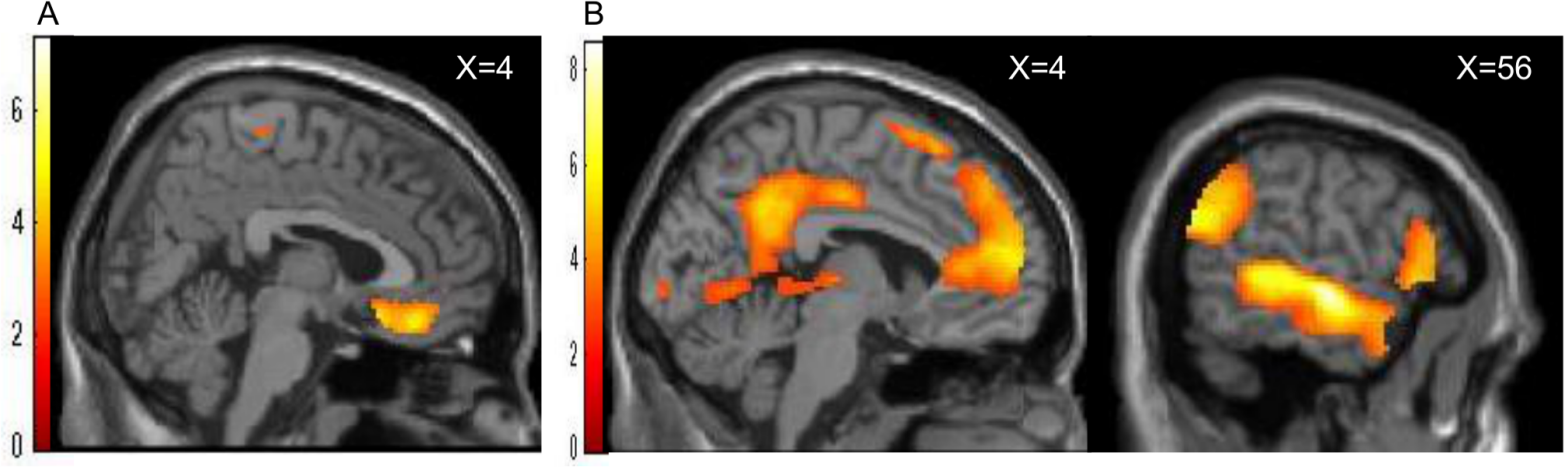
Brain regions indicating ostracism and social support. The brain regions indicating ostracism-induced activation were identified during the social exclusion condition compared to during the social inclusion condition (A) and brain areas indicating social-support-induced activation were identified during the social support condition compared to the social exclusion condition (B).

The entire present sample evidenced several regions of activation during EX compared to IN and during SUP compared to EX. A one-sample *t*-test indicated that, similar to previous studies [17, 36], participants showed significantly increased activity in the right medial prefrontal cortex, including the ventral anterior cingulate cortex (vACC) (x = 4, y = 36, z = -18, *t* = 4.88, cluster size = 1011, *p* < 0.005, Fig 3A, Table 1) and bilateral insula (x = 40, y = -14, z = 18, *t* = 7.30, cluster size = 929, x = -42, y = -18, z = 20, *t* = 6.78, cluster size = 584, *p* < 0.005, Table 1), during EX compared to during IN. Participants also showed significantly increased activity in the bilateral lateral prefrontal cortex (LPFC), bilateral temporal pole (TP), left superior temporal sulcus (STS), bilateral medial prefrontal cortex (MPFC), and bilateral precuneus (Fig 3B, Table 1) during SUP compared to EX. Small volume (ROI) analyses revealed significant activations (FWE-corrected < 0.05, S1 Fig) in the ACC during EX comparing to IN (x = 4, y = 34, z = -16, *t* = 4.64, cluster size = 165, *p* = 0.002, FWE corrected) (Figure A in S1 Fig) and in the DLPFC during SUP comparing to EX (x = -34, y = 24, z = 40, *t* = 5.85, cluster size = 83; *p* < 0.001, FWE corrected) (Figure B in S1 Fig).

**Table 1:** Local maxima of brain activity showing significant social exclusion and social support effects.

Brain Region	BA	*x*	*y*	*z*	Size	*T*	*p*_*uncorrected*_
Exclusion—Inclusion							
R.Insula/Postcentral		40	-14	18	929	7.30	<0.001
L.Insula/Postcentral		-42	-18	20	584	6.78	<0.001
Bi.medial Frontal/ACC	11	4	36	-18	1011	4.88	<0.001
L.Middle/Inferior Occipital/Fusiform	18	-18	-88	-8	160	4.87	<0.001
L.Calcarine		-24	-46	16	273	4.75	<0.001
L.Superior Parietal	3	-18	-36	66	118	4.55	<0.001
L.Middle/Superior Temporal Pole/Inferior~Superior Temporal	38	-38	16	-36	395	4.35	<0.001
L.Postcentral/Precentral	3	-38	-22	48	144	4.28	<0.001
R.Fusiform/Lingual		20	-82	-6	61	4.00	<0.001
L.Fusiform/ParaHippocampal/Inferior Temporal		-28	-28	-16	77	3.46	<0.001
R.Postcentral	4	20	-32	70	48	3.63	<0.001
R.Calcarine/Precuneus		28	-46	14	47	3.44	0.001
R.Postcentral/precentral	3	54	-18	54	101	3.19	0.001
R.Paracentral/L.SMA		-8	-22	60	53	3.16	0.001
Support—Exclusion							
Bi.inferior Frontal/Temporal Pole/Precuneus/Superior Temporal		-46	18	-28	178710	8.56	<0.001
Bi.Medial Frontal/Superior~Middle Frontal/ACC		-14	30	50	6716	6.45	<0.001
R.Caudate		22	16	18	318	4.69	<0.001
L.Cerebrum/Fusiform		-36	-68	-22	228	4.30	<0.001
R.MCC/paracentral		18	-34	48	48	4.28	<0.001
R.Cerebelm/Fusiform		32	-76	-20	38	3.63	<0.001
R.Angular/Inferior Parietal		54	-56	34	171	3.60	<0.001
R.Thalamus		8	-4	0	51	3.34	<0.001
R.Superior Frontal	9	18	30	32	31	3.33	0.001

BA, Brodmann area, Size, cluster size, T, t value of the peak activation within the cluster, Bi, bilateral, L, Left, R, Right, ACC, Anterior Cingulate Cortex, MCC, Middle Cingulate Cortex, SMA, Supplementary Motor Area. Coordinates for the peak voxel are listed as MNI coordinates.

### Regression analysis

**Fig 4:**
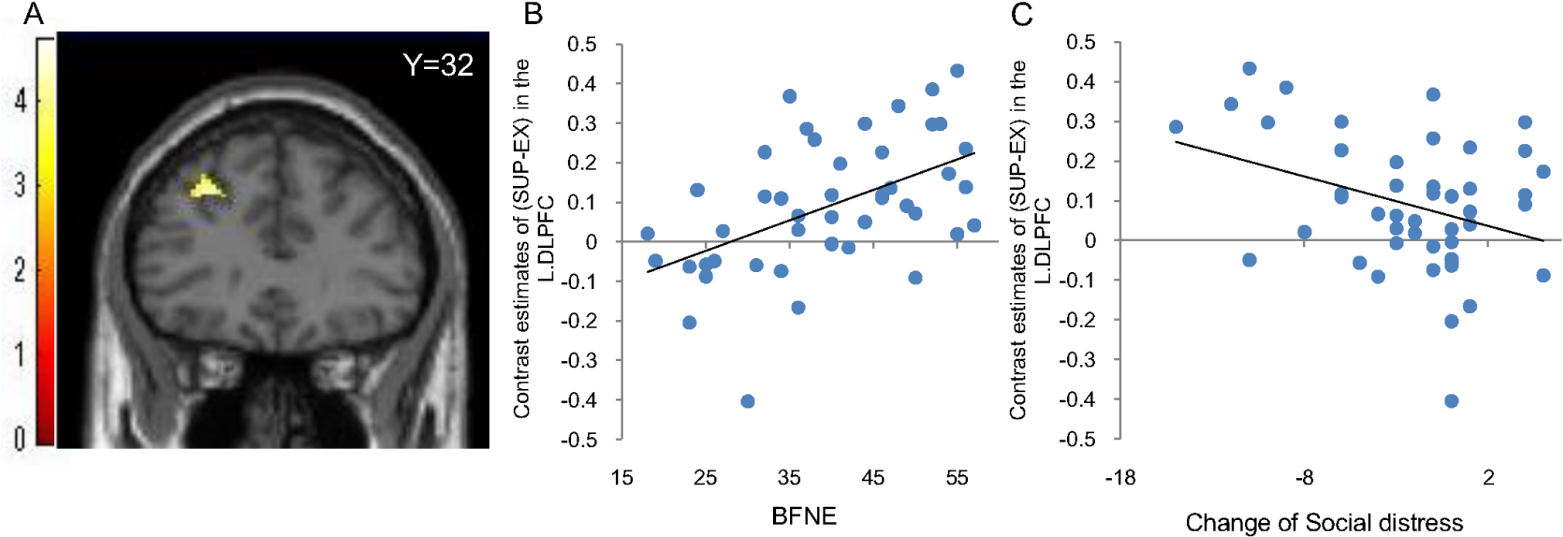
Correlation of the activation in the DLPFC between social anxiety and social distress. The left dorsolateral prefrontal cortex (L.DLPFC), for which significant positive correlation between changes of brain activation and BFNE scores were found (A). To illustrate the correlation between L.DLPFC activation and social anxiety, a scatter plot of the relationship between changes in blood-oxygen-level dependent (BOLD) signals in the L.DLPFC and BFNE scores during the social support condition compared to during the social exclusion condition is presented (B). To illustrate the correlation of L.DLPFC activation and subjective feelings of social distress, a scatter plot of the relationship between changes in blood-oxygen-level dependent (BOLD) signal in the L.DLPFC and subjective feelings of social distress during the social support condition compared to during the social exclusion condition is presented (C). BFNE = the Brief Fear of Negative Evaluation Scale.

We conducted simple regression analyses using BFNE scores to assess possible relationships between social anxiety and social exclusion (EX-IN), as well as social support (SUP-EX) masked by the positive effect of each contrast (inclusive mask threshold was set at *p* < 0.05 uncorrected). Results indicated neither a significant positive relationship nor a significant negative relationship between social anxiety and social exclusion (EX-IN). Moreover, a significant positive relationship between social anxiety and brain activation during social support (SUP-EX) was found for the left DLPFC (x = -28, y = 32, z = 40, t = 4.70, cluster size = 54, *p*_*uncorrected*_ < 0.005, Fig 4A and 4B), whereas no brain region showed a negative relationship between social anxiety and brain activation (SUP-EX). The relationship between social anxiety and DLPFC activation during social support (SUP-EX) survived small volume correction at *p* < 0.05 with FWE correction, within a 20mm-sphere ROI for the DLPFC (x = -28, y = 30, z = 36, *t* = 5.03, cluster size = 17, *p* = 0.004, FWE corrected, S2 Fig) (A and B Figs in S2 Fig).

In order to investigate functions underlying increased left DLPFC activation, we examined the potential relationships between self-reported social distress and brain activation in the left DLPFC during SUP compared to EX. We conducted a correlational analysis to examine changes in social distress during the social support condition compared to the social exclusion condition. We found that there was a statistically-significant negative correlation between changes in subjective social distress and left DLPFC activation (*r* = *-*.364, *p* = 0.018, Fig 4C) during SUP compared to EX.

## Discussion

In this study we examined how social anxiety is correlated with underlying neuronal activities while ostracized people were being provided with emotional support. Results showed that left DLPFC activation was positively correlated with social anxiety. Moreover, changes in left DLPFC activation were negatively correlated with participants’ subjective social distress. This is the first study to report that increased activation in the DLPFC is associated with a decline in subjective social distress in highly socially anxious participants, while they were being provided with emotional support.

The DLPFC is a central regulation area in the brain involved in cognitive control [37–39]. It is known that fear of negative evaluation is one of the core features of SAD. However, positive evaluation is also important for individuals with SAD [40]. We speculated that socially anxious individuals might show increased left DLPFC activity when perceiving supportive messages from others. This is suggestive of an excessive apprehension around stimuli potentially related to evaluation by others, even when the evaluation is positive. The underlying mechanisms of this process, however, remain to be identified. On the other hand, changes in left DLPFC activation were negatively correlated with participants’ subjective social distress, which suggest that their ability to recognize social support remained intact. Since all our participants, including the socially anxious individuals, were university students, they might have been relative well adjusted through effective cognitive control of fears that they experience during their daily lives. It is not clear if this phenomenon could be generalized to different stages of SAD, or whether it could distinguish people with a non-clinical level of social anxiety from a clinical population with SAD. Patients with social phobia have shown decreased DLPFC activation compared to healthy individuals in research using a stressful task [41], social stimuli [42], and reappraisal of negative self beliefs [43]. Hence, in the present study, participants who were socially anxious might have been able to deal with their anxious feelings through increased activation in the DLPFC, without developing symptoms of severe social anxiety. Increased activation in the DLPFC might be involved in facilitating functioning in social situations, in spite of the relatively high social anxiety of these participants. Consequently, DLPFC activation during emotional support might indicate differences between non-clinical and clinical populations of individuals with SAD.

No areas of the brain showed positive or negative relationships with social anxiety, while participants were excluded (EX-IN). Moreover, subjective social distress while participants were excluded from the game, compared to when they were included, did not show a significant relationship with social anxiety. Findings of previous studies examining the effects of social anxiety on ostracism have not been consistent [21, 22, 44]. The present results indicated that neural responses underlying social exclusion were not associated with social anxiety, which could suggest that social isolation itself is not an exceptionally painful experience for people with social anxiety.

The present experimental paradigm demonstrated that when participants were excluded from the ball-tossing game, their subjective social distress increased, which is similar to previous research findings [15–17, 21, 45]. Participants reported that their subjective social distress was the highest when they were excluded from the virtual ball-tossing game (EX), and that their social distress decreased when they received supportive messages (SUP) while they were excluded, compared to when they received no such messages during exclusion (EX). The activation in the vACC was stronger during EX than during IN, which is consistent with previous studies [17, 36], including our own that compared SUP and EX [17] and showed stronger activation in the MPFC, LPFC, TP, and STS. These findings suggest that the present experimental design can successfully replicate the Cyberball paradigm, and therefore that the analysis used in this study was valid. Also, left DLPFC activation was negatively correlated with participants’ subjective social distress, which replicated our previous study [17].

Brain regions that are activated during SUP compared to EX, including the MPFC, temporal pole region, STS, and precuneus, are regions that comprise the theory-of-mind network [46, 47]. Theory of mind refers to the distinction between one’s own thoughts and intentions and those of others [48], as well as to the ability to be aware of mental states of oneself and others [49]. Participants in the present study might have made effective use of theory of mind in order to benefit from socially supportive messages.

There are limitations of this study. It was conducted as a preliminary investigation with non-clinical participants. We considered it appropriate to use a non-clinical population in this study, because social anxiety is considered to lie on a continuum in the general population [3, 5–7]. In addition, it was advantageous to conduct a preliminary study in which the results were not confounded by variations in levels of medication and/or severity of mental disorder. This study demonstrated an association between brain activity and subjective ratings. However, the participants in this study were university students who were likely capable of adequately handling their academic responsibilities and their daily lives, even though some of the participants had several symptoms of SAD. Future studies should sample clinical populations suffering from severe symptoms of SAD that require treatment. Another limitation of this study was that all of the participants were undergraduates and therefore, the results of this study cannot be generalized to younger populations, such as adolescents, or to people older than college undergraduates. Despite the shortcomings of a fixed sequential design, we conducted the Cyberball paradigm in a sequential order, because it was necessary to make participants believe that they were playing catch with real people. Moreover, it was important to prevent participants from anticipating being excluded. If emotionally supportive comments were shown in advance of participants being excluded, this might have aroused participants’ suspicions about the game played via the Internet. From these reasons, we used a fixed sequence, similar to previous studies [16, 17, 36]. As a result, it is possible that the differences in activation between the experimental conditions are confounded by tiredness, or by resignation regarding the experience of being excluded from the social interaction. In the present study, activation of the dACC or insula did not decrease during SUP compared to EX. We interpret this phenomenon, in which social distress is decreased during SUP compared to EX, as the product of increased activation in the DLPFC, rather than decreased activation in the dACC or insula. However, further investigations are required to clarify this account. We used the BFNE as a measure of social anxiety. The BFNE is a brief version of the FNE that assesses an interaction anxiety subtype, which is part of the social anxiety spectrum [26]. Although this is an important aspect of social anxiety [50], future studies should use other commonly used social anxiety scales such as the Liebowitz Social Anxiety Scale [51].

The present study investigated neural response to emotional support provided through positive messages. Given that people with SAD exhibit altered neural reactions to facial expressions suggestive of social rejection [44], future studies should take differences in neural reactions between positive messages and negative messages into consideration, in order to verify anxious reactions to various social interactions.

In summary, the left DLPFC activity during SUP compared to EX was positively correlated with social anxiety as measured by the BFNE. More socially anxious participants showed stronger DLPFC activation when they got emotional support compared to exclusion without emotional support. Social anxiety was associated with an increased BOLD signal in the left DLPFC when individuals were offered positive social support while being excluded.

## Supporting Information

S1 Fig Brain regions indicating social ostracism and social support, according to ROI analyses, using brain regions indicated in an earlier study [17]. (TIF)

S2 Fig Relationships between brain activity and social anxiety, according to ROI analyses, using brain regions indicated in an earlier study [17]. (TIF)
