# Supplementary figures and images for: fMRI Study of Social Anxiety during Social Ostracism with and without Emotional Support
Source: PLoS One. 2015 May 22;10(5):e0127426. doi: 10.1371/journal.pone.0127426 (PMC4441506; doi:10.1371/journal.pone.0127426)

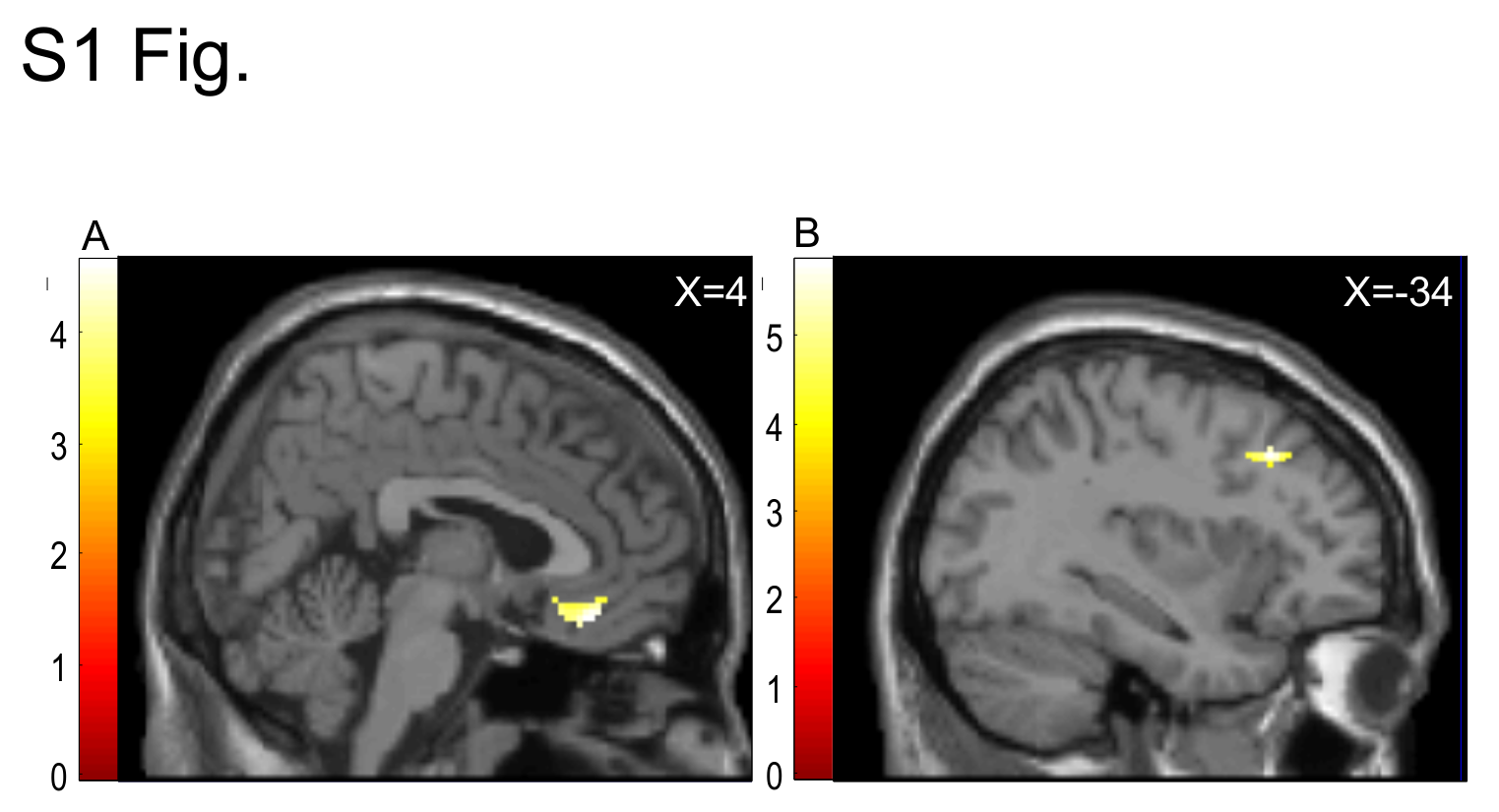

Supplement: S1 Fig — (TIF) [file pone.0127426.s001.tif]

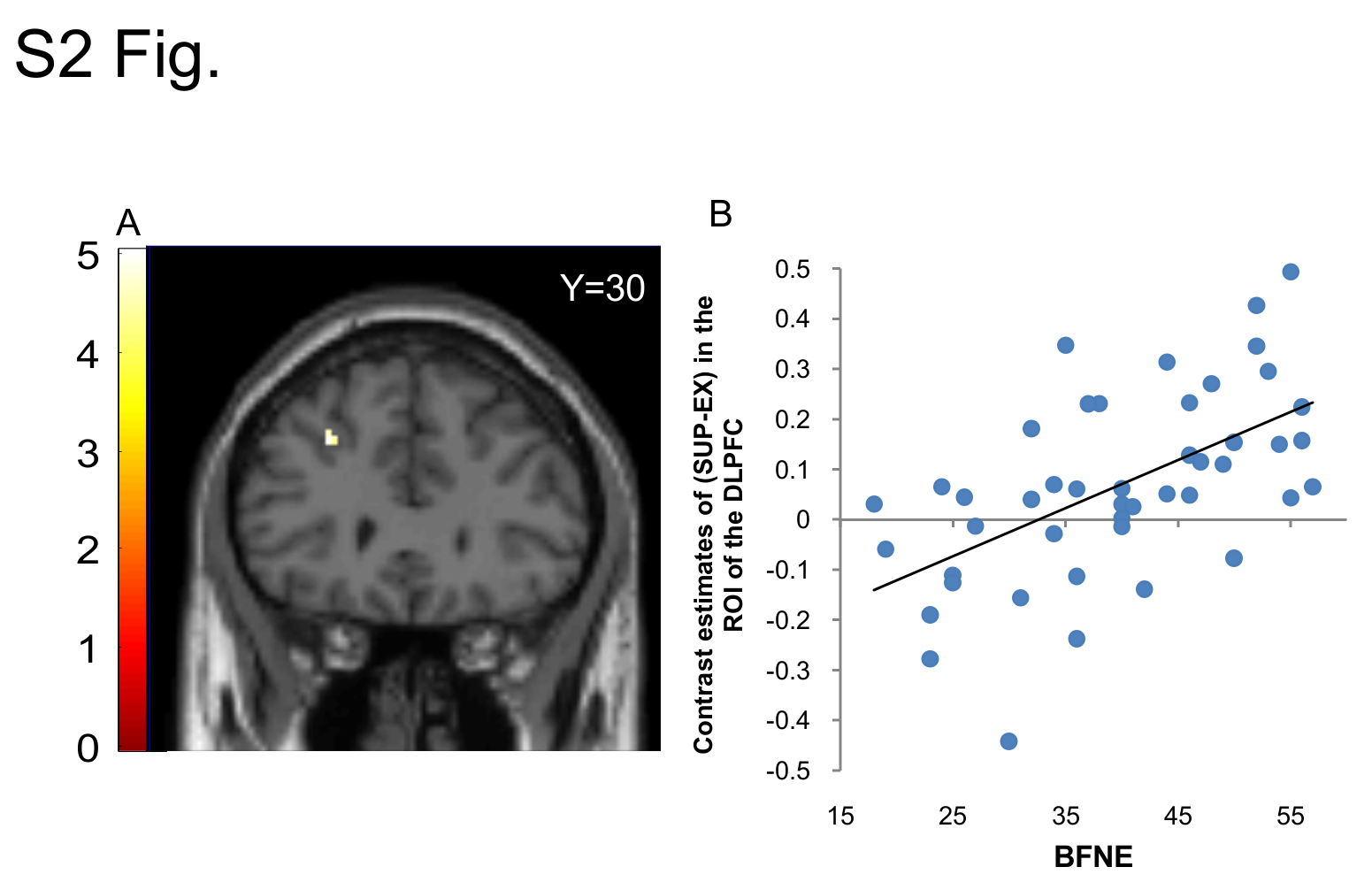

Supplement: S2 Fig — (TIF) [file pone.0127426.s002.tif]
